# Connectivity of bacterial assemblages along the Loa River in the Atacama Desert, Chile

**DOI:** 10.7717/peerj.9927

**Published:** 2020-10-01

**Authors:** Ana Zárate, Cristina Dorador, Ruben Araya, Mariela Guajardo, July Z. Florez, Gonzalo Icaza, Diego Cornejo, Jorge Valdés

**Affiliations:** 1 Doctorado en Ciencias Aplicadas mención Sistemas Marinos Costeros, Universidad de Antofagasta, Antofagasta, Chile; 2 Laboratorio de Complejidad Microbiana y Ecología Funcional, Instituto Antofagasta & Centro de Bioingeniería y Biotecnología (CeBiB), Universidad de Antofagasta, Antofagasta, Chile; 3 Humedales del Caribe colombiano, Universidad del Atlantico, Barranquilla, Colombia; 4 Departamento de Biotecnología, Facultad de Ciencias del Mar y Recursos Biológicos, Universidad de Antofagasta, Antofagasta, Chile; 5 Laboratorio de Microbiología de Sedimentos, Departamento de Acuicultura, Facultad de Recursos del Mar, Universidad de Antofagasta, Antofagasta, Chile; 6 Doctorado en Genómica Integrativa y Centro GEMA, Facultad de Ciencias, Universidad Mayor, Santiago, Chile; 7 Centro i mar and CeBiB, Universidad de Los Lagos, Puerto Montt, Chile; 8 Departamento de Ciencias Farmacéuticas, Universidad Católica del Norte, Antofagasta, Chile; 9 Chair of Technical Biochemistry, Technische Universitāt, Dresden Dresden, Germany; 10 Laboratorio de Sedimentología y Paleoambientes, Instituto de Ciencias Naturales A. von Humboldt, Facultad de Ciencias del Mar y de Recursos Biológicos, Universidad de Antofagasta, Antofagasta, Chile

**Keywords:** Desert river, Bacterial assemblages, Fragile environment, Core taxa, Network connectivity

## Abstract

The Loa River is the only perennial artery that crosses the Atacama Desert in northern Chile. It plays an important role in the ecological and economic development of the most water-stressed region, revealing the impact of the mining industry, which exacerbate regional water shortages for many organisms and ecological processes. Despite this, the river system has remained understudied. To our knowledge, this study provides the first effort to attempt to compare the microbial communities at spatial scale along the Loa River, as well as investigate the physicochemical factors that could modulate this important biological component that still remains largely unexplored. The analysis of the spatial bacterial distribution and their interconnections in the water column and sediment samples from eight sites located in three sections along the river catchment (upper, middle and lower) was conducted using 16S rRNA gene-based Illumina MiSeq sequencing. Among a total of 543 ASVs identified at the family level, over 40.5% were cosmopolitan in the river and distributed within a preference pattern by the sediment substrate with 162 unique ASVs, while only 87 were specific to the column water. Bacterial diversity gradually decreased from the headwaters, where the upper section had the largest number of unique families. Distinct groupings of bacterial communities often associated with anthropogenic disturbance, including Burkholderiaceae and Flavobacteriaceae families were predominant in the less-impacted upstream section. Members of the Arcobacteraceae and Marinomonadaceae were prominent in the agriculturally and mining-impacted middle sector while Rhodobacteraceae and Coxiellaceae were most abundant families in downstream sites. Such shifts in the community structure were also related to the influence of salinity, chlorophyll, dissolved oxygen and redox potential. Network analyses corroborated the strong connectivity and modular structure of bacterial communities across this desert river, shedding light on taxonomic relatedness of co-occurring species and highlighting the need for planning the integral conservation of this basin.

## Introduction

Lotic systems represent a link between terrestrial and marine environments, playing an important role in the movement and cycling of energy, nutrients and other materials (Battin et al., 2008) which are crucial for ocean productivity. They provide a large array of ecological processes, as well as acting as reservoirs of biodiversity (Besemer et al., 2013). Aquatic microbial communities are particularly diverse and widely recognized as being central driving force of biogeochemical cycles and biodegradation-biotransformation of chemical compounds relevant to maintenance of ecosystem health and balance (Bai et al., 2014).

To date, many studies of riverine microbial ecology have largely focused on major urban and floodplain river systems across the world including the Elbe River (Böckelmann et al., 2000), Ebro River (Ruiz-González et al., 2013), Mississippi River (Staley et al., 2015), River Thames basin (Read et al., 2015) and others. Similarly, because microbial processes in river ecosystems vary widely, a growing body of work has provided evidence that bacterial communities in rivers can show specific spatial variation in structure and function reflecting from shifts in physicochemistry, land use and hydrological properties across catchments (Zoppini et al., 2010; Zeglin, 2015; Gao et al., 2017).

Water availability is regarded as one of the most important factors that constrain biological activity in deserts (Zeglin et al., 2011). As such desert rivers represent ecological arteries with important implications for patterns and process of the dependent biotic communities but in general, there is a paucity of information on rivers located at the driest end of the hydrological spectrum and their biota (Jacobson & Jacobson, 2013). Moreover, microbial dynamics in desert rivers and streams are poorly understood (Bunn et al., 2006), relatively few studies have investigated microbial community structure in ephemeral river systems (Fluke, González-Pinzón & Thomson, 2019; Sánchez-Montoya et al., 2020). This is unfortunate, given the vulnerability of these ecosystems under a climatic change scenario which will result in increased stress (Prieto, 2015; Williams, 2017).

Therefore, there is a pressing need to characterize microbial processes in order to determine the level of ecosystem functioning and hence resilience, in order to confront ongoing desertification (Adhikari et al., 2019; Cavicchioli et al., 2019). In this context, Fluke, González-Pinzón & Thomson (2019) proposed that sediment-water interactions should be explicitly considered in water quality management, protection, and improvement efforts of the Arid River of the Rio Grande, which receives the treated wastewater, agricultural return flows, and urban stormwater runoff near Albuquerque in New Mexico.

The Loa River is the longest (440 km) river system in Chile. Flowing from East to West, rising in the Andes, and crosses the Atacama Desert and finally flows into the SE Pacific Ocean (Pell et al., 2013). The catchment is in the Antofagasta Region in northern Chile. This, the most water-stressed region in Chile, holds a marked competition for water, largely to support the demands of copper and other metals for mining, agriculture, as well as other anthropogenic activities (Bugueño et al., 2014). Pestle et al. (2019) recognizes that life (human or otherwise) would be impossible in such a setting were it not for the presence of the Loa River, yet flow in the Loa River is often very reduced in some sections with a high rate of water extraction for mining purposes (lower than 1 m^3^/s), with marked impacts on biodiversity and human populations (Camacho, 2012; Jordan et al., 2015).

The location and environmental conditions of the Loa River add to its distinctiveness: furthermore, it supports many endemic taxa (e.g., *Telmatobius dankoi*, *Telmatobius halli*, *Heleobia stimpson*, *Liolaemus paulinae*, *Microluphus maminesis* and *Pseudalopex griseus*) which display adaptation to life in the most arid region in the world (Aravena & Suzuki, 1990; Collado, Valladares & Méndez, 2016). Unfortunately, few biological surveys have been conducted in the Loa River, and little is known about this fragile river ecosystem (Palma et al., 2013).

Water resources in the Loa River are managed in pseudo-independent sections, referred to as the Upper, Middle, and Lower Loa. These sections can be distinguished according to their different physicochemical, geological properties and highly variable heavy metals concentrations, including As, Ni, Hg and Cu (Romero et al., 2003; Bugueño et al., 2014; Jordan et al., 2015). This management acts a block to the implementation of integrated management of the catchment and the capacity to deal with issues such as water demand, pollution, flooding, droughts, and the interaction between surface and underground water (Peña, 2018).

Unlike some other aquatic habitats located in the Atacama Desert, such as the shallow lakes and saline wetlands at high altitude and have repeatedly been shown to harbor unique microorganisms (e.g., Dorador et al., 2009; Dorador et al., 2013; Fernandez et al., 2016) the Loa River has received little scientific attention. To the best of our knowledge, no studies have examined bacterial community structure and function throughout the basin. As such, little is known about the spatial variability of microbial diversity at local scales and potential interaction with physicochemical heterogeneity of this aquatic ecosystem.

Considering these issues, this study examines the following questions: Are there differences in microbial community composition across the catchment? If so, which factors influence community structure? Is there is a microbiological continuum along the Loa River, and does the hydrology facilitate bacterial connectivity across different sectors? Overall, this study explored spatial variation in microbial diversity in water and sediments in this desert river across a marked altitudinal gradient (from 3300 m a.s.l to sea level), using Illumina sequencing of the 16S rRNA gene from environmental samples. The contributions of sediment to water microbial diversity were also evaluated. In addition, we used network analysis to identify interconnected river microbial assemblages. Understanding the community composition and diversity of microbial communities along physiochemical gradients will help identify ecological processes through which microbes change along the aquatic continuum to adapt to local extreme conditions (Doherty et al., 2017).

## Materials and Methods

### Site description, sampling and environmental variables

**Figure 1:**
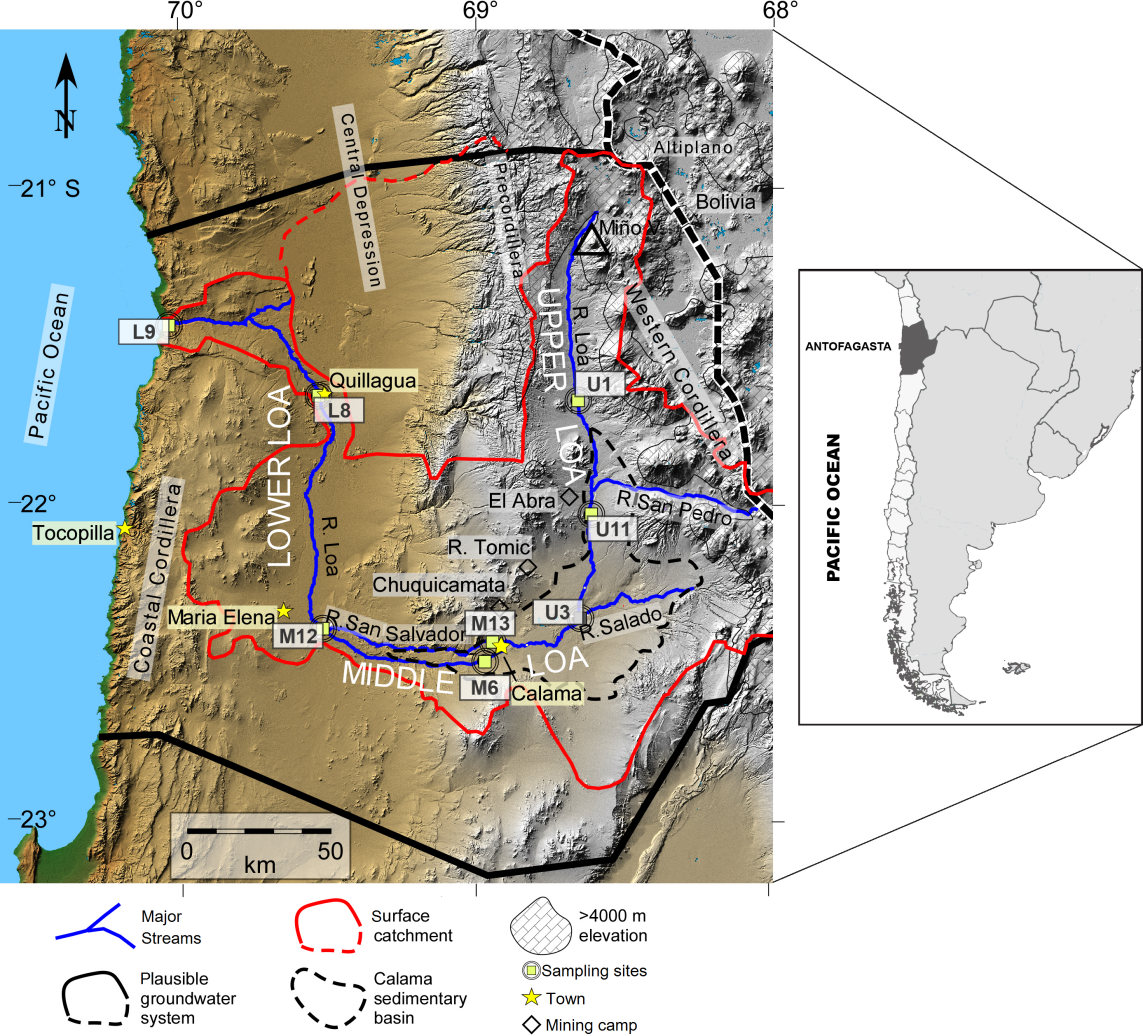
Map of the Loa River Basin located in northern Chile and location of sampling sites. Base is a digital elevation model in which tan colors show elevations below ∼2,500 m, gray indicates higher elevation. The formally defined boundaries of the Loa surface water basin are located at U (dividing upper, middle and lower Loa). Map created by Teresa Jordan. Used with the Geological Society of America (GSA) permission. View original article DOI:10.1130/GES01176.1.

The Loa River basin (Fig. 1) originates at the foot of the Miño Volcano (5651 m a.s.l), located in the Antofagasta Region of northern Chile). It is the longest river in the country (440 km) and the catchment extends across an area of 33,570 km^2^. Crosses the Atacama Desert from east to west all the way to the Pacific Ocean (see Fig. 1), representing the main source for industrial mining (e.g., the Chuquicamata copper mine), agricultural, and domestic water supply (Valdés-Pineda et al., 2014).

The extremely arid conditions in the region favor high evaporation rates and high salinity. As such, all rivers in the region are temporal or endorrheic except for the Loa River, which is permanently exorheic, with flow varying seasonally in the range of 0.5–3 m^3^/s. The river drains a catchment rich in metalloids, including arsenic (concentrations of up to 27 mg As L^−1^ are attributed to natural and human sources, notably the contribution from El Tatio Geothermal field) as well as copper, boron, chloride, sulfate and other elements (Romero et al., 2003; Bugueño et al., 2014).

The main tributaries of the Loa are the San Pedro, El Salado, and San Salvador rivers, which originate in the high Andes (Pell et al., 2013). As such, the Loa basin has a rain-dominated hydrologic regime, with flows typically increasing during the Austral summer (January and February) reflecting increased precipitation during the so-called Altiplanic Winter, when flows can be up to four orders of magnitude above baseflows (Jordan et al., 2015). However, this aquatic system is a critical water resource in a water-scarce region with an intense water usage pattern (Bugueño et al., 2014).

We sampled at eight representative sites distributed systematically along the Loa River (Fig. 1). Sites were selected to address issues that affect all sections of the river, such as pollution (from agriculture, mining, urban inputs). Sites were also selected that are identified within the Chilean National water network database for monitoring, established by the General Water Authority (DGA; its acronym in Spanish) which allowed us to cover a wide range of spatial heterogeneity that offers a unique opportunity to investigate how biotic and abiotic factors modulate the structure of bacterial communities in different sections of the river.

We sampled three sites from the upstream sector of the catchment during the austral spring of 2014: Lequena (U1), Output Reservoir Conchi (U11), and the river before the junction with the Salado River (U3), three in the middle sector (La Finca (M6), the river after the junction with the San Salvador River (M12), and the origin of the San Salvador River (M13), and two downstream (the river before Quillagua (L8) and the river mouth (L9)). Sampling sites U1, U3, and U11 have a mean flow of 1.2 m^3^/s and are characterized by the dominance of aquatic vegetation (algae, shrubs, and grasses) within the stream and on the two banks of the river. Supporting bofedales (local name for wetlands) and unique peatlands (Villagrán & Castro, 1997). Mean flow at the sampling sites M6, M12, and M13 are 0.25 m^3^/s. Human activities in this sector are associated with mining (e.g., the Chuquicamata, Radomiro Tomic, and El Abra mines), agriculture, and the chemical industry (Valdés-Pineda et al., 2014).

The hydromorphology of the Lower sector of the Loa catchment (L8 and L9) has been significantly altered since the early 1900s, following to the construction of three dams, Sloman, Santa Fe and Santa Teresa. These have not only regulated flow, but have also affected the natural sediment flux from the Andes to the Pacific Ocean (Aravena & Suzuki, 1990; Bugueño et al., 2014). The Quillagua oasis (L8) is a natural wetland with abundant aquatic life and was the main agricultural area in the Loa (Pell et al., 2013). Site L9 is the point where the river enters the Pacific Ocean but the water flow is too low (0.44–0.60 m^3^/s) considering the total available annual recharge of the Loa surface. The groundwater hydrological basin is 6.4 m^3^/s, which is attributed to evapotranspiration and consumptive water use (Romero et al., 2003; Jordan et al., 2015), thereby there is no major estuarine zone.

At each of eight sites (Fig. 1), we collected surface water (approximately depth from the surface 5 cm) and sediment samples. At each sampling site, three 1 L replicates of river water were collected manually into sterilized amber Boston bottles from the riverbank. These samples were pre-filtered firstly through 3.0 µm pore size polycarbonate filters (GSWP, Millipore) and then through 0.22 µm pore size to collect particle-attached and free-living bacteria, respectively, with an electric vacuum pump, connected to a portable electric generator in the field. Each filter was cut into half immediately following filtration in conical sterile tubes and both fractions were analyzed. The filtration time was no longer than 30 min, and the filters were replaced when the filtration speed slowed down due to the clogging.

Sediment samples were collected using a clean plastic scoop. A sterile spatula was used to transfer an aliquot of the scooped sample into a sterile 50 mL Falcon™ tube. All samples were transported on ice (4 °C) to the laboratory and stored at −20 °C prior to DNA extraction. At each sampling site we determined water temperature, salinity, conductivity, pH, redox potential, dissolved oxygen (DO) and chlorophyll concentrations *in situ* with a CTD Sea Bird 19 Plus multiparameter probe. Site locations and physical data are presented in Table 1.

**Table 1:** Coordinates and water properties of the sampling sites.

**Sampling sites**	**Code**	**UTMCoordinates zone**		**Altitude m a.s.l**		**Temperature (°C)**	**Electrical conductivity (µS/cm)**	**pH**	**ORP (mV)**	**DO(mg/L)**	**Chlorophyll (µg/L)**	**Salinity**
		**East**	**North**									
Loa River in Lequena	U1	535264	7604060	3320	Mean	11.3	1205.7	8.61	264.12	74.5	15.4	0.60
					Min	11.2	1197.0	8.55	254.00	74.1	5.50	0.60
					Max	11.4	1215.0	8.67	272.00	75.0	28.6	0.61
					SD	0.09	9.02	0.06	9.21	0.45	11.9	0.01
Output Reservoir Conchi	U11	539132	7564018	2950	Mean	7.5	3256.2	8.16	263.92	60.6	9.08	1.71
					Min	7.4	3228.0	7.90	204.00	58.6	5.20	1.69
					Max	7.5	3286.0	8.48	306.00	62.2	13.4	1.72
					SD	0.03	29.0	0.29	53.29	1.86	4.12	0.02
Loa River before junction with Salado River	U3	536036	7527190	2640	Mean	13.7	3873.6	7.78	262.07	92.4	39.7	2.06
					Min	13.7	3857.0	7.63	196.00	87.2	33.4	2.05
					Max	13.7	3889.0	7.94	315.00	96.6	46.7	2.07
					SD	0.02	16.0	0.16	60.58	4.76	6.69	0.01
Loa River in La Finca	M6	501127	7511218	2050	Mean	14.9	9061.1	9.12	182.62	70.4	5.24	5.10
					Min	14.9	8983.0	9.09	179.00	69.2	4.40	5.05
					Max	14.9	9147.0	9.16	186.00	71.6	6.00	5.15
					SD	0.02	82.3	0.04	3.51	1.19	0.80	0.05
Loa River after junction with San Salvador River	M12	444892	7524183	1230	Mean	14.4	9565.9	8.87	156.73	68.6	7.22	5.40
					Min	13.4	9211.0	8.46	140.00	63.7	6.10	5.19
					Max	15.1	9987.0	9.25	174.00	72.4	8.50	5.66
					SD	0.85	392.2	0.40	17.01	4.44	1.21	0.24
Loa River before Agricultural Area of Quillagua	L8	443087	7605780	790	Mean	19.2	10096.7	8.29	95.47	104.3	12.2	5.72
					Min	19.2	10049.0	8.29	91.00	103.5	11.6	5.69
					Max	19.3	10142.0	8.30	100.00	105.2	12.7	5.74
					SD	0.05	46.6	0.01	4.50	0.84	0.55	0.03
Loa River Mouth (where enters Pacific Ocean)	L9	390985	7630340	1.0	Mean	19.3	18542.7	8.86	66.58	104.0	6.49	11.02
					Min	19.3	18477.0	8.86	63.00	103.5	6.10	10.98
					Max	19.3	18610.0	8.86	70.00	104.4	6.90	11.07
					SD	0.03	66.5	0.00	3.50	0.44	0.40	0.05
Source of the San Salvador River	M13	504145	7518431	2250	NM	NM	NM	NM	NM	NM	NM	NM

**Notes.**

All measurements made with a Seabird CTD probe 19 Plus. Except sampling site M13 where data could not be reliably m 3 easured due to site conditions (mud and garbage).

### DNA extraction, PCR amplification and sequencing

DNA was extracted from individual water and sediment samples using the DNA Isolation Kit *PowerSoil* ™ (MoBio Laboratories, CA. USA), according to the manufacturer’s instructions. Quantity and purity were tested in a NanoDrop 2000 (Thermo Scientific, Wilmington, DE, USA) and quality was checked by 0.8% p/v agarose gel electrophoresis. A mixture of DNA was made from the true replicate sample, resulting in 16 samples, divided into two sets of 8 samples for each substrate type (water/sediment) that were finally sequenced.

The hypervariable V1–V3 regions of bacterial 16S rRNA genomic sequences were PCR-amplified with universal primers (27 F mod 5′—AGR GTT TGA TCM TGG CTC AG—3′; 519 R mod bio 5′—GTN TTA CNG CGG CKG CTG—3′), with a barcode attached to the forward primer, through 30 cycles of PCR using the HotStarTaq Plus Master Mix Kit (Qiagen, USA). The PCR conditions were as follows: 94 °C for 3 min, followed by 28 cycles of 94 °C for 30 s, 53 °C for 40 s and 72 °C for 1 min, after which a final elongation step at 72 °C for 5 min was performed. After amplification, PCR products were checked in 2% agarose gel electrophoresis. Multiple samples of the same original sample were pooled together in equal proportions based on their molecular weight and DNA concentrations. Pooled samples were purified using calibrated Ampure XP beads. Then the pooled and purified PCR product was used to prepare a DNA library by following Illumina TruSeq DNA library preparation protocol. The read length was 300 bp, and the reads were both forward and reverse. Sequencing was performed at MR DNA (http://www.mrdnalab.com, Shallowater, TX, USA) on a MiSeq following the manufacturer’s guidelines. Sequencing results were obtained as fasta-qual and pipeline files.

### Bioinformatic and statistical analysis

#### Processing and data analysis

Miseq 16S rRNA raw reads dataset (forward and reverse) were processed using the DADA2 pipeline (Callahan et al., 2016) and R package version 1.9.1 in RStudio 1.1.463 (R 3.5.2). Sequences were trimmed and filtered using truncLen (220, 120 bp) parameter based on their quality profile (plotQualityProfile) and maximum expected errors (maxEE). Using learnErrors() function parameter amplicon sequence variants (ASV) were evaluated and then derreplicated to combined all identical into one. Then running the dada function, sequencing errors were removed, at last forward and reverse reads were paired merge (mergePairs). Chimeras were removed using the removeBimeraDenovo function. Finally, ASVs taxonomic assignment were conducted by assignTaxonomy function against the SILVA 132 database (99% clustering), chloroplast was removed from the data table.

Microbial composition was described from phylum to genus level. First, the relative abundance of each ASV within each sample was calculated, then the ASVs were sorted in descending order according to their relative abundance. Raw sequence data produced in this study was deposited in ENA-SRA database accession number PRJEB28365.

#### Comparison of microbial communities

Alpha (α-diversity) and beta diversity (*β*-diversity) were estimated using QIIME (De Filippis et al., 2013). Calculations for the Shannon, Evenness, and Simpson diversity indices were performed using R (Fig. S1). Further, a rarefaction curve was constructed by a random sampling of the sequences, and a rank abundance curve was developed to reflect the pattern of distribution of ASVs.

Statistical analysis of microbial relative abundance was carried out at the level of genus, based on ASV abundance and taxonomic phylogenetic information (Lozupone & Knight, 2007).

Correlations between community structure and environmental variables were explored by using Canonical Correspondence Analysis (CCA) with the software package CANOCO v4.5 for Windows, and the tool CANODRAW was used to visualize the CCA (ter Braak & Smilauer, 2002). A variance inflation factor ≤10 was used to reduce the multi-collinearity among predictive variables. Furthermore, the overall variation in bacterial community was depicted by non-metric multidimensional scaling analysis (NMDS) (Kenkel & Orlóci, 1986) based in Bray-Curtis distance and tested the effects of river sections on microbial assemblages by permutational multivariate analysis of variance (PERMANOVA) in the “vegan” package of R (Dixon, 2003). ANOSIM and SIMPER analyses was performed in PRIMER v6 (Clarke & Gorley, 2006) to identify those ASVs that most characterized the bacteria community composition of Loa River at each sampling sites or that mostly contributed to the differences observed. Cut-off value was restricted to 60%.

#### Network analysis

Associations between the microbial communities within river samples were examined by calculating all possible Pearson rank correlations between bacterial genera using the Otu.association command from Mothur v.1.39.5. Only correlations with pairwise Pearson correlations (*r* ≥ 0.95) and statistically significant (*p* ≤ 0.01) were considered to be a valid interaction event. It is important to highlight that the alternative similarity metrics (include Spearman, Pearson and Kendall correlation) may be appropriate for particular datasets, but in this study, the Pearson correlation coefficient was considered most robust for identifying association networks. In addition, Popović (2015) indicated that Spearman’s rank correlation coefficient is equivalent to Pearson correlation on ranks, even when the first have fewer assumptions. When assumptions about heteroscedasticity and normality were violated, the data were transformed to square-root. However, Pearson correlation was not used to test the relationship between environmental factors and ASVs in this work.

Putative keystone ASVs were determined using the Betweenness Centrality metric, defined as the number of shortest paths going through a node (BC> 0); the increasing BC values indicate a greater contribution of nodes to the network structure (Ma et al., 2016). The connectivity of the network was evaluated using the Closeness Centrality (CC) (Blondel et al., 2008), which is defined as the number of steps required to access all other nodes from a given node (Ma et al., 2016). Therefore, nodes with highest betweenness centrality value, indicate the relevance of a node as capable of holding together with communicating nodes, were recognized as “gatekeepers” or keystone taxa in co-occurrence networks (Widder et al., 2014) .

In order to describe the topology of the resulting network, a set of metrics (i.e., average path length, network diameter, average node connectivity, graph density, cumulative degree distribution, clustering coefficient, and modularity) were calculated. Finally, the network structure was characterized using the Fruchterman–Reingold layout (Fruchterman & Reingold, 1991), and explored/visualized data with the interactive platform Gephi (Bastian & Heymann, 2009).

## Results

### Physicochemical parameters of the Loa River

The physical and chemical characteristics of the samples are shown in Table 1. Although at all sampling sites conductivity was high for a river Conductivity values increased along the river from 3300 m a.s.l to the mouth. In the Upper river section (U1, U11 and U3), the samples show the lowest conductivity (1217 to 3874 µS/cm). While in the Middle-Low section (M6, M12, L8 and L9), the value increased from 5931 µS/cm to 18541 µS/cm.

Dissolved oxygen concentrations varied considerably (58.5 –105.1 mg/L) between sites, with higher altitude sites (>1200 m) showing lower concentrations (58.5 to 87.2 mg/L) and highest values being observed from sites nearest to the river mouth (103.4 to 105.1 mg/L).

Chlorophyll concentrations were related to the characteristics of each sampling site, with highest concentrations being recorded found at sites U1, U3 and L8 stations (11.33 mg/L, 38.84 g/L and 25.73 mg/L, respectively). These sites were characterized by abundant aquatic vegetation made up of instream algae and macrophytes and riparian vegetation. pH values ranged from 7.7 and 9.1 among the eight sampling sites, the highest value was recorded at station M6 and the lowest value at U3. On average, water temperature increased with distance from source from 11.25 °C to 19.28 °C, except for station U11 where temperatures averaged 7.46 °C. It should be noted that this parameter depends on the time of day when the sample was taken.

### Spatial distribution of bacterial assemblages along the Loa river

After quality filtering and discarding singletons, chimeras and chloroplast sequences, a total of 473266 reads were obtained, with a mean of 29579 sequences per sample with an average length of 452 bp. These represented a total of 7935 bacterial ASVs with 99% similarity, for a total of 16 samples sequenced from the water column and sediment, sampling at eight sites located along the Loa River (Fig. 1).

Phylogenetic characterization identified 50 phyla, 129 classes, 319 orders, 543 families and 1056 genera at both substrates. Based on the phylogenetic classification, ASVs were assigned to Proteobacteria, Bacteroidetes, Epsilonbacteraeota, Planctomycetes, Firmicutes, Chloroflexi and Actinobacteria, as the most abundant taxa (relative abundance >1%), that made up 87% of the diversity in datasets.

**Figure 2:**
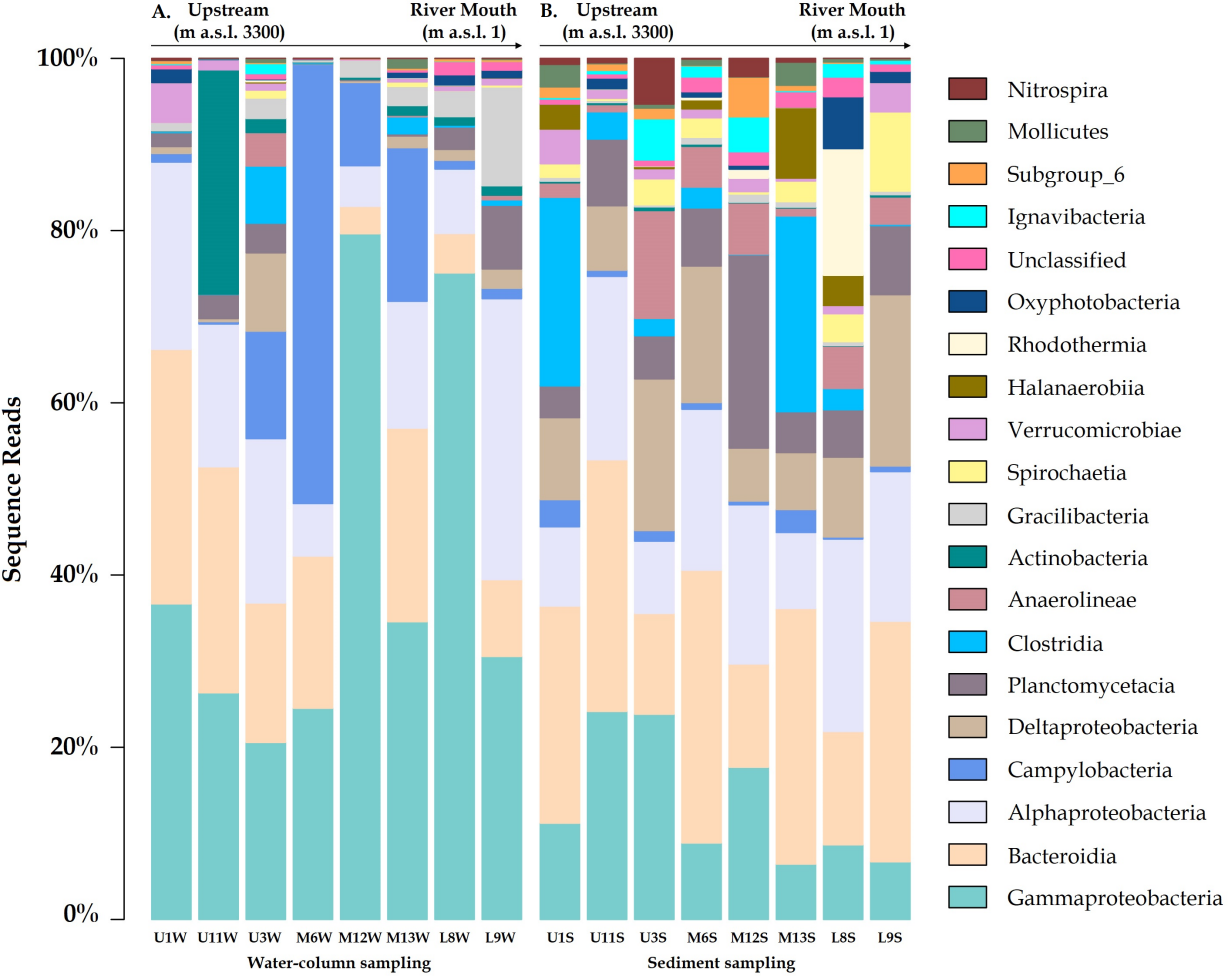
Community compositions profile at the class level for the (A) water column and (B) sediment samples from each site around Loa River. Representative classes of relative abundances more than > 1%.

**Figure 3:**
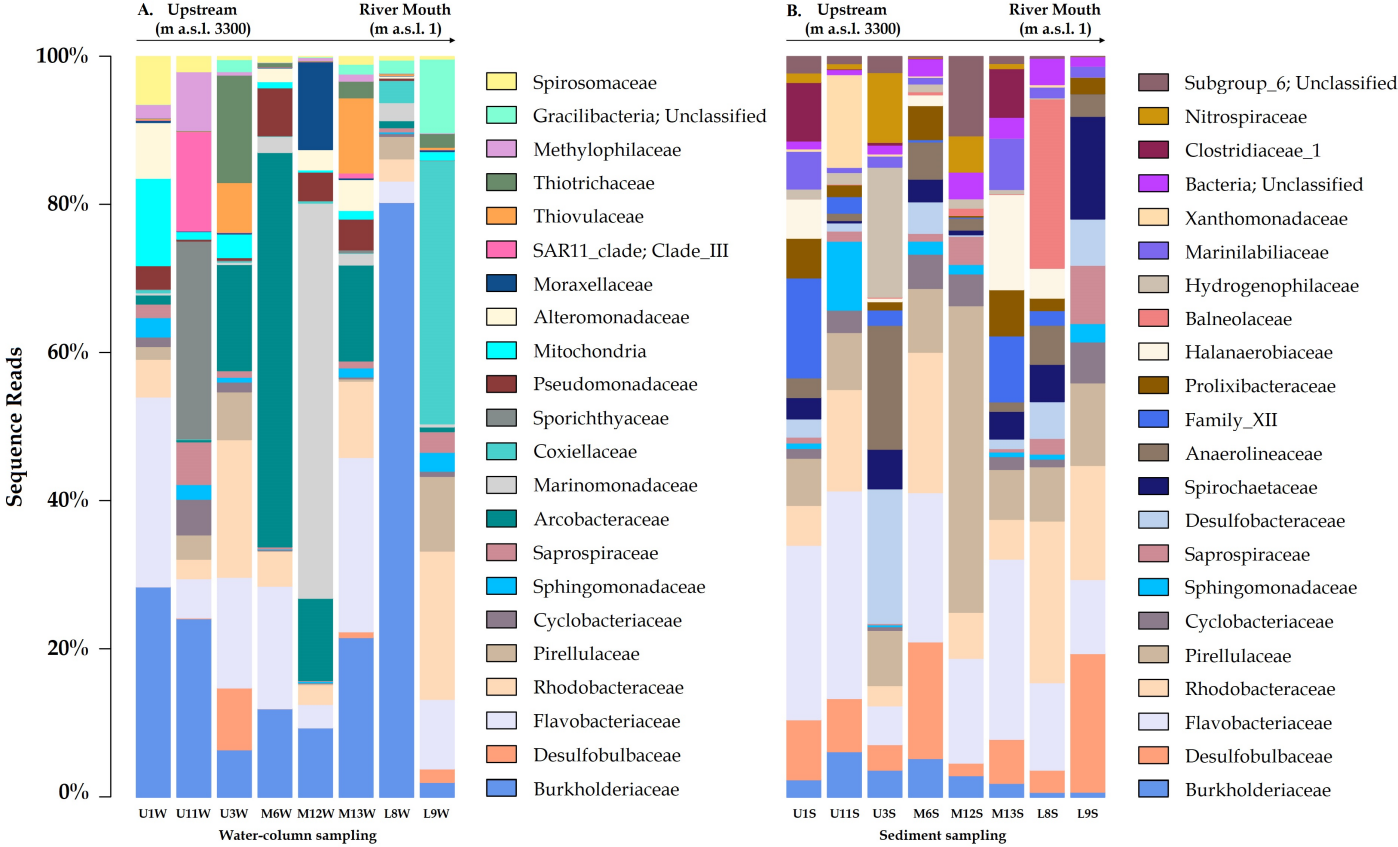
Variation bacterial community composition at all sampling sites along the Loa River. (A) Distribution at family level in the water column and (B) sediment samples (relative abundance >1% at least in one sample).

The microbial communities differed between the water column and sediment samples. The water column contained taxa groups related to Gammaproteobacteria (Burkholderiaceae family with a relative abundance of 19.4%), Campylobacteria (Arcobacteraceae - 5.7%) and Actinobacteria, represented by Sporichthyaceae family (1.9%, especially in the U11 sampling site; Fig. 2A). Meanwhile, Bacteroidia (Flavobacteriaceae), Deltaproteobacteria (Desulfobulbaceae) and Planctomycetacia (Pirellulaceae), were most abundant taxa in the sediment samples, accounted for 10.4%, 5.3% and 3.6% of total ASVs, respectively (Figs. 2–3B).

Relative abundance of Arcobacteraceae and Marinomonadaceae families increased in the water column of the mid-sector of the Loa catchment, specifically in M6, M12 and M13 sampling sites, with a strong differentiation between the upstream and downstream. Similarly, Burkholderiacea, Rhodobacteraceae and Coxiellaceae were the major families in a downstream site (L8 and L9), which included agricultural lands and the desert habitat where the river enters the Pacific Ocean.

Furthermore, water bacterial communities from the Loa River, with the diversity identified at the genus-level, comprised mainly of *Arcobacter (4.9%), Hydrogenophaga (4.3%), Marinomonas (3.2%) and Flavobacterium (3.1%) members. Meanwhile, Fusibacter, Halanaerobium, Actibacter and Thiobacillus* exhibited a higher abundance in the sediment (1.0%). The results also revealed that the less abundant bacteria in both substrates (<0.7% of the total sequences) were unevenly distributed. However, the high percentage of unclassified ASVs at genus level in the sediment samples did not allow providing detailed information about the substrates at this level.

### Relationship between α- and *β*-diversity at different sampling sections of the Loa River

Our results showed that α-diversity was slightly higher in sediment samples (compared to water) for a variety of metrics, including observed number, Chao1, and Shannon (Table 2). Sites located in the upper part of the catchment displayed the highest bacterial diversity. Conversely, sites in the middle sector of the catchment, the lowest α-diversity was observed in water samples. However, among the whole bacterial community, different sampling sectors did not vary significantly (*p* > 0.05). ANOSIM confirmed that the similarity of microbial communities was greater in the different sampling sectors (Global R: 0.07; *P* > 0.1) than in the substrate type (Global R: 0.561; *P* = 0.001).

**Figure 4:**
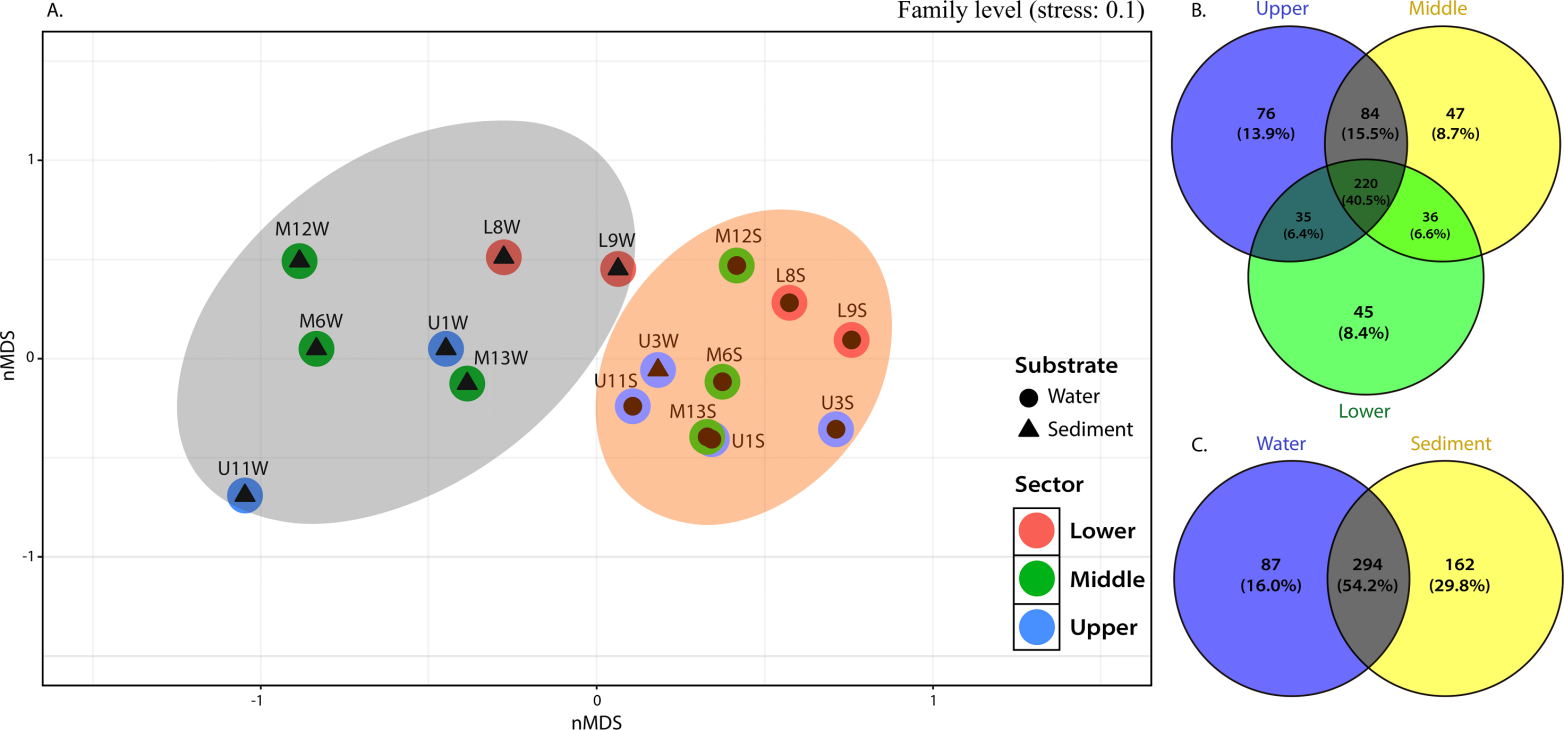
nMDS (A) from Bray-Curtis illustrated the dissimilarity between sampling sites across the river. Venn diagram indicate unique and shared ASVs at family level among the (B) different sections and (C) substrate types in the Loa river.

The NMDS plots also showed marked differences in bacterial community structures, with a clear separation of water samples from sediment samples (Fig. 4A). PERMANOVA was then performed, and the results further corroborated that bacterial communities in the water column were significantly different from those in the sediment (p=0.001). Considering the relative abundance of ASVs in some sediment samples from the middle and upper sectors dominated by greater communities, as evidenced by their higher evenness or homogeneity (Inverse Simpson), it was suggested that sediment samples captured most of the bacterial taxa, resulting in higher diversity.

**Table 2:** Alpha diversity measures of the bacterial communities in different sampling sites and subtrates types along the Loa River (Upper, Middle and Lower section). Observed ASVs, Chao1, Shannon diversity and the inverse Simpson index.

			**Diversity indices values**				
**Substrate**	**Site**	**Sequence number**	**Observed**	**Chao1**	**Shannon**	**InvSimpson**	
Water column	U1W	32862	856	864.8	5.93	138.0	
	U11W	28686	280	280.0	4.39	43.8	
	U3W	57128	799	801.1	5.95	155.4	
	M6W	46941	402	407.0	3.92	11.1	
	M13W	29541	333	335.5	5.24	101.4	
	M12W	49750	342	343.2	4.49	28.1	
	L8W	31723	767	767.3	3.85	6.53	
	L9W	8348	896	899.0	5.59	40.9	
Sediment	U1S	19124	617	617.4	5.80	157.0	
	U11S	20678	882	887.0	6.24	286.1	
	U3S	25894	791	791.7	6.22	296.6	
	M6S	26368	750	759.7	5.97	144.2	
	M13S	24250	676	676.0	5.88	127.7	
	M12S	31583	824	828.1	6.40	455.2	
	L8S	16818	690	690.4	5.82	162.5	
	L9S	23572	619	619.0	5.65	125.6	

**Notes.**

Number of total sequences: 473266.

This observation was supported by the distribution gradient of the core, defined as those common to most samples in this study and shared bacterial families, which illustrated the extensive overlap between the upper and middle sections (15.5% ASVs) and less overlap between upper-lower and middle-lower sections with ASVs of 6.6% and 6.4%, respectively (Fig. 4B). The upper section had the largest number of unique families (see Fig. 4), followed by the middle and lower sections. This is reflected in the placement of bacterial communities in the upper sector, incorporating 76 ASVs (13.9%) until 47 (8.7%) and 45 (8.4%) unique ASVs of bacterial assemblages in the middle and lower sections of the river, respectively (Fig. 4B).

From 543 ASVs identified at family level, over 220 (representing 40.5%) were cosmopolitan to the three sampling sections of the river and distributed within a clear preference pattern by the sediment matrix. That is, about 162 families (29.8%) were unique ASVs in the sediment, while only 87 (16.0%) were specific to the column water (Fig. 4C).

Next, SIMPER analysis attributes 75.0% of dissimilarity recorded mainly to Arcobacteraceae, Burkholderiaceae and Flavobacteriaceae, which generated a contribution greater than or equal to 0.9%. Members of Desulfobulbaceae, Marinomonadaceae, Pirellulaceae, Anaerolineaceae, Pseudomonadaceae, Halanaerobiaceae, Alteromonadaceae, Flavobacteriaceae, Prolixibacteraceae, Spirochaetaceae and Hydrogenophilaceae contributed more than 0.5% for the community dissimilarity.

### Influential physicochemical factors on bacterial communities along the river

**Figure 5:**
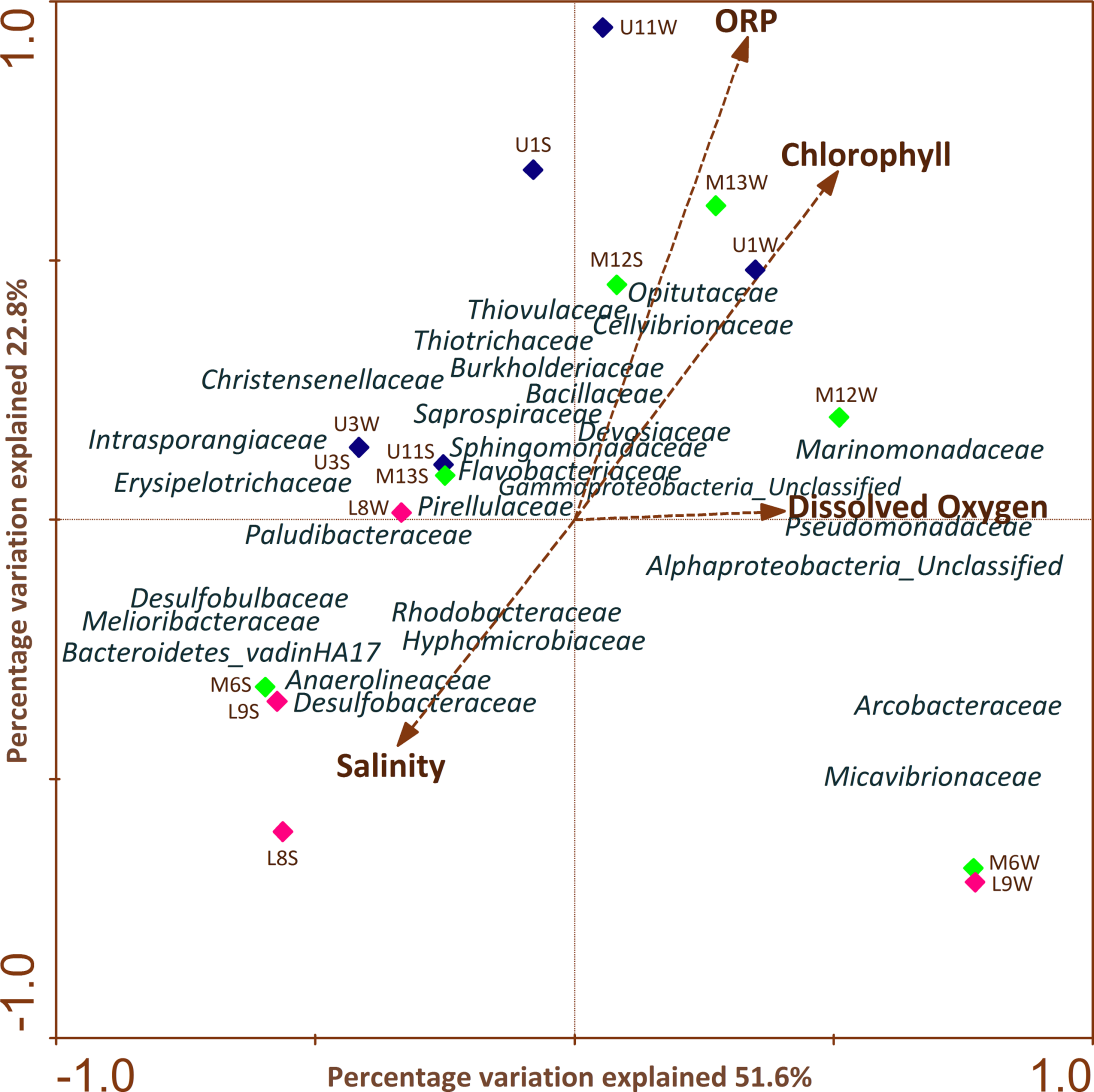
CCAs of bacterial community structure from column water and sediment samples using environmental variables. The length of the arrow represents degree of correlation with presented axis. Correlations between environmental variables and the first two CCA axes are represented by the lengths and angles of the arrows (environmental-factor vectors). Only abundant bacterial ASVs (>1%) are shown in the triplot.

CCA (Fig. 5) highlighted the differences in profiles obtained from samples of water and sediment and their relationships with different physiochemical parameters. It is important to note that the relation of a particular parameter with the community composition and the samples is provided by the length of a physicochemical parameter arrow in the ordination plot. The cumulative percentage of the variance of the species-environment indicates that the first and second canonical axis explained 51.6% and 22.8% of this variation, respectively. The remaining axes accounted for less than 16% of the total variance and therefore were not considered. It was determined that the first axis was positively correlated with all environmental variables, except with salinity (Fig. 5).

CCA analysis showed that ORP, chlorophyll and salinity were the main variables explaining variation in bacterial ASV composition. Sphingomonadaceae, Opitutaceae, Thiovulaceae, Flavobacteriaceae, Cellvibrionaceae, Saprospiraceae, Thiotrichaceae, Marinomonadaceae, Pseudomonadaceae, Devosiaceae, Bacillaceae were positively correlated with ORP, chlorophyll and DO mainly in the upper and middle section of the river. Meanwhile, Paludibacteraceae, Hyphomicrobiaceae, Rhodobacteraceae, Anaerolineaceae, Desulfobacteraceae, Desulfobulbaceae, Melioribacteraceae and Bacteroidetes_vadinHA17 families were strongly and negatively correlated with salinity in sediment samples across the different sectors.

### Bacterial networks interactions

**Figure 6:**
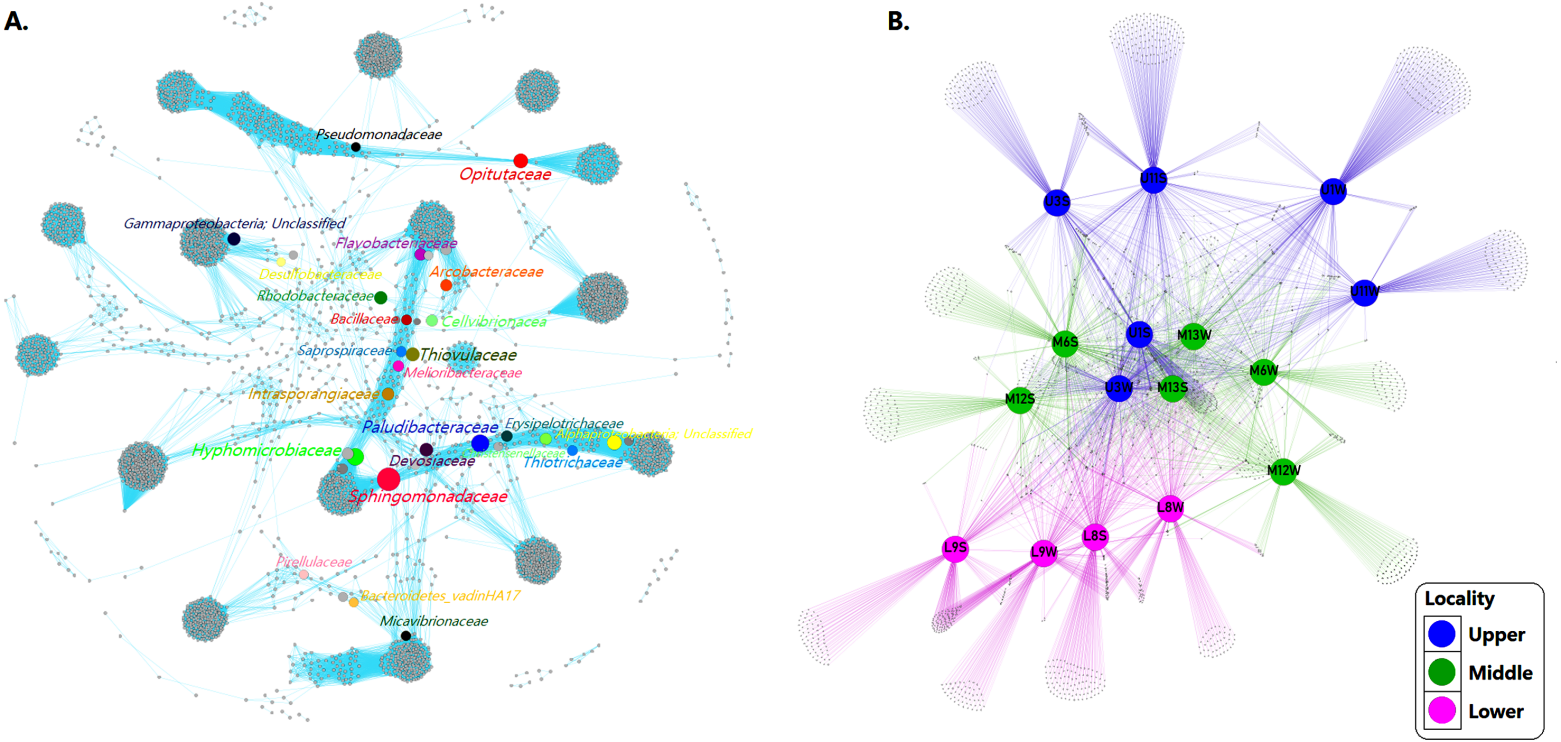
Networks of microbial communities along the Loa River. (A) The co-occurrence network interactions. (B) Bacterial patterns of presence/absence of bacterial community across sampling sites. The connection stands for a strong Pearson’s (*p* ≥ 0.95) and significant (*P* ≤ 0.01) correlation. The nodes (circles) represented unique sequences in the data sets and the sizes of each one is proportional to the relative abundance; the color of nodes shows different taxonomies. Here, color denotes the gradient following the shape of the river (dividing upper, middle and lower sector).

We inferred a co-occurrence network based on correlation relationships and *p*-values, adjusted with the Fruchterman-Reingold layout algorithm which allowed the visual identification of a clustered topology, expressed as a set of “closely nodes” (that is, taxa richness with average path length 1843) connected predominantly by blue edges (average degree 90447; Fig. 6A). These relationships between taxa shaped the network as a modular structure of bacterial communities (modularity index of 0.901; see Table 3).

**Table 3:** Network features and taxonomy of keystone taxa of the Loa River. ASVs with highest betweenness centrality overall dataset were selected as the keystone taxa.

**ASVsID**	**Network features**				**Taxonomy**			
	**Betweenness centrality**	**Closeness centrality**	**Degree**	**Modularity class**	**Phylum**	**Class**	**Order**	**Relative abundance (%)**
Sphingomonadaceae	17484.6	0.308	184	13	Proteobacteria	Alphaproteobacteria	Sphingomonadales	1.10
Paludibacteraceae	9233.9	0.343	41	19	Bacteroidetes	Bacteroidia	Bacteroidales	0.30
Hyphomicrobiaceae	8792.2	0.261	190	13	Proteobacteria	Alphaproteobacteria	Rhizobiales	0.60
Opitutaceae	6786.7	0.374	106	56	Verrucomicrobia	Verrucomicrobiae	Opitutales	0.40
Thiovulaceae	6159.6	0.317	39	16	Epsilonbacteraeota	Campylobacteria	Campylobacterales	0.70
Rhodobacteraceae	5856.0	0.197	8	16	Proteobacteria	Alphaproteobacteria	Rhodobacterales	5.90
Unclassified	5583.6	0.300	235	46	Proteobacteria	Gammaproteobacteria	Unclassified	0.50
Intrasporangiaceae	5266.9	0.300	39	16	Actinobacteria	Actinobacteria	Micrococcales	0.02
Christensenellaceae	5070.7	0.358	48	19	Firmicutes	Clostridia	Clostridiales	0.20
Flavobacteriaceae	4869.4	0.373	169	16	Bacteroidetes	Bacteroidia	Flavobacteriales	9.30
Arcobacteraceae	4704.8	0.298	25	16	Epsilonbacteraeota	Campylobacteria	Campylobacterales	5.00
Unclassified	4704.8	0.2977	212	19	Proteobacteria	Alphaproteobacteria	Unclassified	0.60
Cellvibrionaceae	4518.3	0.233	9	16	Proteobacteria	Gammaproteobacteria	Cellvibrionales	0.20
Erysipelotrichaceae	4499.4	0.352	39	19	Firmicutes	Erysipelotrichia	Erysipelotrichales	0.10
Melioribacteraceae	4208.4	0.446	41	16	Bacteroidetes	Ignavibacteria	Ignavibacteriales	0.10
Saprospiraceae	3824.7	0.316	39	16	Bacteroidetes	Bacteroidia	Chitinophagales	1.20
Thiotrichaceae	3812.2	0.548	57	19	Proteobacteria	Gammaproteobacteria	Thiotrichales	1.00
Bacillaceae	3740.5	0.402	33	16	Firmicutes	Bacilli	Bacillales	0.10
Devosiaceae	3591.4	0.291	29	19	Proteobacteria	Alphaproteobacteria	Rhizobiales	0.20
Micavibrionaceae	3417.1	0.397	166	52	Proteobacteria	Alphaproteobacteria	Micavibrionales	0.40
Pseudomonadaceae	3066.5	0.458	50	0	Proteobacteria	Gammaproteobacteria	Pseudomonadales	1.10
Bacteroidetes_vadinHA17	3031.4	0.293	11	52	Bacteroidetes	Bacteroidia	Bacteroidales	0.50
Desulfobacteraceae	2829.0	0.255	14	46	Proteobacteria	Deltaproteobacteria	Desulfobacterales	1.50
Pirellulaceae	2779.6	0.383	14	8	Planctomycetes	Planctomycetacia	Pirellulales	3.90

Based on the modularity class, the network analysis of the total bacterial community contained 3,966 nodes and 358,713 edges. Fourteen groups of interactive genera were observed (Fig. 6A). The inner associations among each subcommunity indicated 3966 strongly connected components, in contrast with just 43 weakly connected components.

At the community level, core ASVs affiliated to the network including Flavobacteriaceae (9.3%), Rhodobacteraceae (5.9%), Arcobacteraceae (5.0%), Pirellulaceae (3.9%), Desulfobacteraceae (1.5%), Saprospiraceae (1.2%), Sphingomonadaceae (1.1%), Thiotrichaceae (1.0%) and Pseudomonadaceae (1.1%) were dominant families in both sediment and water samples community. The network also displayed taxa with less relative abundance and high values BC (see Table 3) such as Intrasporangiaceae (0.01%), Melioribacteraceae (0.1%), Erysipelotrichaceae (0.1%), Bacillaceae (0.1%), Devosiaceae (0.2%), Christensenellaceae (0.2%), Cellvibrionaceae (0.2%), Paludibacteraceae (0.3%), Micavibrionaceae (0.4%), Opitutaceae (0.4%), Bacteroidetes_vadinHA17 (0.5%), Hyphomicrobiaceae (0.6%) and Thiovulaceae (0.7%).

However, despite the bacterial communities varied among sites probably because the habitat heterogeneity, the patterns of presence/absence network showed that mainstem communities were spatially homogeneous, revealing a considerable overlap of ASVs among the different sampling sites. Indicating that the bacterial assemblages in the river present a well-defined core and strongly interconnected through the sectors but differ significantly (*p* = 0.001) between sediment and water samples (Fig. 6B).

## Discussion

This study provides the first insights into the strong spatial connectivity of bacterial assemblages that flow across the Loa River, which is the principal aquatic artery in the Atacama Desert (Pell et al., 2013), where the water is the main limiting resource for many organisms and their ecological processes (Warren-Rhodes et al., 2007); however, there are water stress associated with this arid environment that is extremely fragile and sensitive to climate changes and human disturbances, including over-extraction for mining and agriculture, as well as urban and agricultural discharges (Valdés-Pineda et al., 2014; Bull et al., 2016).

### Shift of bacterial assemblages with the substrate types along the river

We detected a total of 50.5% known bacterial phyla in the Loa River (50 out of 99 phyla reported) (Parks et al., 2018). This diversity is comparable to the Liu Xi River Reservoir in China, where 48 phyla were found (Wang et al., 2012). In contrast, the bacterial community of the Upper Mississippi River included 32 phyla (Staley et al., 2013). However, Payne et al. (2017) identified 37 phyla of free-living bacteria and 49 phyla in the particle-associated fraction collected from the Mississippi River.

Such diversity suggests that the Loa River could provide an “extreme local reservoir” that helps harbor the major prokaryotic diversity among the driest terrestrial biome of the world (Bull et al., 2016), playing a significant role in shaping bacterial communities and their dispersal into the stream, which are considered relevant to understanding the coupling of biogeochemical cycles and energy fluxes between the riverine and Pacific Ocean ecosystem (Velimirov et al., 2011; Aufdenkampe et al., 2011).

The results of this study also revealed that bacterial diversity gradually decreased from the headwaters to the lower reaches, especially in the water column where the Shannon index decreased (upstream: 5.95; middle: 3.92; lower: 3.85). Nevertheless, these changes were not significant (*p* > 0.05) between different sampling sectors. Therefore, it was assumed that α-diversity in the Loa River remained relatively constant across the catchment, with similar distribution trends in diversity. This trend has previously been observed in the Amazon River, where microbial communities were shown did not vary spatially over the 675 river kilometers (Doherty et al., 2017).

In contrast, significant differences (PERMANOVA, *p* = 0.001) in the bacterial assemblages were observed between water and sediment samples, exhibiting higher relative abundance in sediment than water samples. It is noteworthy that although the connection between the microbial community structure and spatial variation in rivers has been widely investigated (Böckelmann et al., 2000; Read et al., 2015; Adhikari et al., 2019), water and sediments together have rarely been explored (Lu et al., 2016).

In this respect, our results are consistent with previous studies in the Fenghe River (Lu et al., 2016), the Yellow River estuary (Wei et al., 2016), and the Santa Ana River (Ibekwe, Ma & Murinda, 2016), where greater prokaryotic diversity was observed in sediments compared to water, and bacterial communities in sediments were clearly distinct from those of the overlying water. These studies also reported that Proteobacteria phylum included more than 50% of the total number of bacterial sequences. However, a previous study on sediments of the Camarones River located approximately 250 km further from the north in the Atacama Desert, identified and isolated Alphaproteobacteria, Betaproteobacteria and Gammaproteobacteria in both water and sediments (Azua-Bustos & González-Silva, 2014). To our knowledge, the current study represents the first report of microbial community structure in the Loa River.

It is not surprising to find that the sediment entrapped the majority of the bacterial communities because this matrix, commonly documented as a relatively stable habitat, serves as a reservoir of nutrients and organic matter (Staley et al., 2015; Wei et al., 2016). Moreover, stream sediments in desert environments are habitats that likely promote unique patterns of microbial diversity (Zeglin et al., 2011), thus playing an important role in the formation of microbial mats that can help microorganisms assemble and resist environmental changes (Read et al., 2015; Lu et al., 2016).

Furthermore, patterns observed in this study are very similar to those reported in previous studies, which bacterial communities were dominated by Proteobacteria (46.3%), followed by Bacteroidetes, Epsilonbacteraeota, Actinobacteria, Patescibacteria, Planctomycetes, Firmicutes, Verrucomicrobia, Chloroflexi and Acidobacteria, accounting more than 93% of bacterial diversity. Such phyla are regarded as typical freshwater bacteria and are commonly found in large/urban rivers around the world (e.g., De Oliveira & Margis, 2015; Read et al., 2015; Adhikari et al., 2019).

Chen et al. (2018) and Hou et al. (2019) investigated the bacterial communities in riparian sediments and found that Proteobacteria, Firmicutes, Bacteroidetes, Acidobacteria, Actinobacteria, Chloroflexi, Verrucomicrobia, and Planctomycetes were predominant, comprising of the ranged of 85% to 93% of the total sequence data along a large-scale longitudinal gradient. In addition, comparative studies of the community structure of aquatic bacteria in urban rivers yielded similar results, indicating that the most abundant genes belonged to Proteobacteria, Bacteroidetes, Actinobacteria, Acidobacteria, Firmicutes, Chloroflexi and Verrucomicrobia (Kolmakova et al., 2014; Doherty et al., 2017).

### Factors shaping the bacterial assemblages

The overall findings showed that bacterial assemblages of the Loa river were correlated with changes in physicochemical properties, including salinity, DO concentration, ORP, and chlorophyll. This is agreement with previous studies from several river systems reporting how shifts in the diversity and distribution of the bacterial community composition were strongly predicted by environmental variables (Ortega-Retuerta et al., 2013; Zhang et al., 2019).

Our results also showed the possible impacts of the anthropogenic activities like discharge on physicochemical variables measured, which in turn, could explain the dissimilarities observed in bacterial assemblages at upstream and downstream observed (Kolmakova et al., 2014; De Oliveira & Margis, 2015; Read et al., 2015), especially in the middle to the downstream section of the river, where a decline in diversity (Drury, Rosi-Marshall & Kelly, 2013; Jani et al., 2018), as well as distinct groupings of bacterial families that are often associated with nutrient pollution (e.g., Arcobacteraceae, Flavobacteriaceae, Rhodobacteraceae, Burkholderiaceae, Sphingomonadaceae and Pirellulaceae) were observed. For example, Arcobacteraceae including Campylobacterales, was particularly dominant in untreated and river samples obtained from a wastewater discharge area (Bai et al., 2014). Similarly, Rhodobacteraceae and Pirellulaceae were among the most dominant groups in the most urbanized sites along a highly impacted stream (Roberto, Van & Leff, 2018).

Banerjee, Schlaeppi & van der Heijden (2018) reported that various members of Burkholderiaceae, more specifically of Burkholderiales order, largely distributed in the water column of the upper and lower sections of the river, were consistently identified as keystone taxa in different ecosystems, such as desert shrubland, Antarctic lakes, contaminated soil, sediments of Amazonian lakes and other rivers. The elevated abundance of Flavobacteriaceae members may reflect the high inputs of organic matter in the middle sector of the river because this family is mainly particle-associated and strongly dependent on the availability of organic matter (Newton et al., 2011). Likewise, Desulfobacteraceae, has been reported as family typically associated with regular organic loads (Saravanakumar et al., 2012).

In the present study, the middle areas of the river experienced higher degrees of discharge associated with mining effluents (Chuquicamata and El Abra mines), as well as agricultural and urban wastewater discharges (Romero et al., 2003; Valdés-Pineda et al., 2014). In this context, a marked increase in Sporichthyaceae from the Actinobacteria phylum obtained from water samples at site U11 was observed. The family Sporichthyaceae includes species adapted to both impacts associated with urban wastewater (Newton & McLellan, 2015) and waters contaminated by acid mine drainage (Ettamimi et al., 2019). This can support the notion that local conditions in different sections of the Loa River could influence the structure of the microbial community (Payne et al., 2017).

### Co-occurrence network of the bacterial assemblages in the Loa River

Desert bacterial communities are recognized as being unstable and vulnerable to change (Wang et al., 2019). However, the increased of water and nutrient availability afforded by the water supply from a river, provides increased scope for interactions between different species (Zeglin et al., 2011). Therefore, with regard to the study of bacterial assemblages, our co-occurrence analysis provided insights into structural variation and interactions between bacterial communities in the water and sediment along the Loa River.

The network analysis identified common ASVs as strongly interconnected mainstream community assemblages along the river that were spatially homogeneous. Among total of 543 ASVs identified at the family level, over 220 (40.5%) were ubiquitous in all three river sections and distributed with a clear preference for the sediment matrix. The core bacterial assemblage, present throughout the study area (Staley et al., 2013; De Oliveira & Margis, 2015; Savio et al., 2015), was dominated by members of the Proteobacteria, including mainly Sphingomonadaceae, Hyphomicrobiaceae and Rhodobacteraceae and Bacteroidetes (Paludibacteraceae, Flavobacteriaceae and Saprospiraceae).

In addition, recent studies of fluvial microbial co-occurrence networks identify gatekeepers affiliated with Saprospiraceae and Sphingomonadaceae (Widder et al., 2014). Sphingomonadaceae are strict aerobic chemoheterotrophs, frequently reported to be found in soils and eutrophic waters and on corals and plant surfaces (Vaz-Moreira, Nunes & Manaia, 2011). Moreover, this family has been found in habitats that contain high proportions of xenobiotic and recalcitrant aromatic compounds in various studies reported (Zhou et al., 2016).

The core communities, found in the Loa River were also comprised of numerically inconspicuous families including Intrasporangiaceae, Opitutaceae, Hyphomicrobiaceae, Melioribacteraceae, Bacillaceae, Devosiaceae, Paludibacteraceae, Cellvibrionaceae, Micavibrionaceae. The major ecological role of these taxa in different aquatic ecosystems has remained unclear. Therefore, their characterization, along with a spatial disturbance gradient, may help us understand the bacterial community functioning in the Loa basin. Nevertheless, keystone taxa are increasingly recognized to regulate the functioning in terrestrial and aquatic environments (Jousset et al., 2017).

Our findings concur with those of previous studies in which keystone species that are typically not the most abundant/dominant species, were identified (Zhao et al., 2016; Hu et al., 2017). Thus, it is important to take into account that the abundance of a species is not the best determinant of its contribution to the community (Lynch & Neufeld, 2015).

The Loa River did not exhibit clear fragmentation between different sampling sections. The higher bacterial diversity reported in this study likely increases network interconnections and the robustness of the community at a larger scale. A possible explanation for these observations is that the flashy nature of the hydrological profile of the Loa River system, where large water flow occurs during rain-dominated hydrological regimes in December to-March, may drive the homogenization of microbial assemblages in the river system (Hu et al., 2017). These findings are important as they show that the strong connectivity of the bacterial assemblages in a desert river has been maintained by major hydrologic modifications since the early 1900s to support mining and other human activities (Bugueño et al., 2014). Such connectivity could enhance the survival and mutually beneficial interactions between microbes (Graham et al., 2017), in depauperate environments such as desert rivers, where nutrients are limiting.

This is a first attempt to characterize the microbial community of the Loa River. We are aware that future work must include a temporal component to improve understanding of the temporal microbial dynamics in multiple hydrological periods. In addition, we recognize the need to include other specific components of the microbiome, namely archaea and eukaryotes, whose interactions can modulate the entire environmental, biological function under natural conditions, and the need to examine the influence of a wider range of physicochemical factors such as nutrients and heavy metals on microbial community. This would provide insights into the likely ecological consequences of the ongoing deterioration of this important aquatic system, as well as the global ecological risk assessment of desert environments in the context of anthropogenic changes.

## Conclusions

In northern Chile, wherever you look is the ocean or the desert and the horizon is a challenge to the survival itself. Here, the Loa River is the connection between both immensities. This study has shed light on the bacterial community structure and the strong connectivity of their assemblages to spatial scale along this fluvial system. We found that local bacterial communities interact with environmental aspects such as salinity, ORP and chlorophyll, and the structures of these communities are influenced by significant effects of substrate type along the river. Is very relevant that this desertic river might have validated the findings of pioneering investigations on riverine microbes with a core microbiome, which are key players in maintaining the ecological stability of the ecosystem. Suggesting that the Loa River could provide an “extreme local reservoir” that helps harbor the major prokaryotic diversity among the driest terrestrial biome of the world. However, is particularly concerning high abundances of bacterial communities associated with polluted water, which poses high ecological risks in the middle section of the river possibly due to mining activities and sewage discharge from Calama city in this sector, thus causing strong environmental stresses, which is predicted to increase with the ecological effects of climate change. This latter is predicted to increase with the ecological effects of climate change, affecting the ecological integrity of the river. Thus, a detailed exploration of variation in microbial community structure over different temporal as well as their diversity-function relationship will be the next important step in the unraveling of microbial ecology in the Loa River.

##  Supplemental Information

10.7717/peerj.9927/supp-1 Supplemental Information 1 Alpha diversity measurements of the bacterial composition in different sections and substrate types along the Loa River From left to right, Observed, Chao1 species richness estimates, Shannon index and Inverse of Simpson index. Values are mean ± SD.

## Additional Information and Declarations

### Data Availability

The following information was supplied regarding data availability: The sequencing data are available at the European Nucleotide Archive: PRJEB28365.
