# Supplementary figures and images for: Connectivity of bacterial assemblages along the Loa River in the Atacama Desert, Chile
Source: PeerJ. 2020 Oct 1;8:e9927. doi: 10.7717/peerj.9927 (PMC7533063; doi:10.7717/peerj.9927)

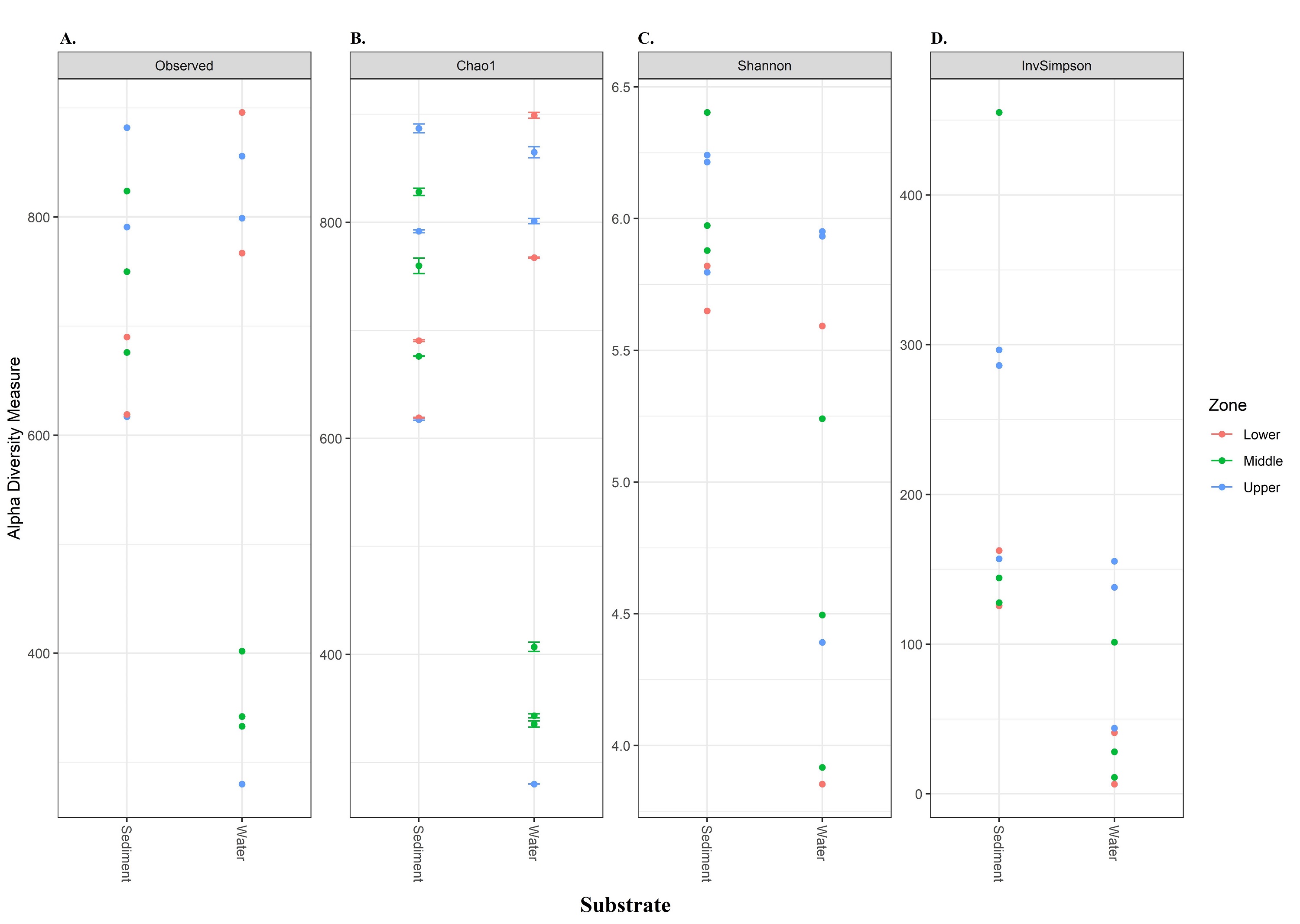

Supplement: Supplemental Information 1 — From left to right, Observed, Chao1 species richness estimates, Shannon index and Inverse of Simpson index. Values are mean ± SD. [file peerj-08-9927-s001.jpeg]
